# Serial multiple mediating role of coping style and anxiety in the relationship between life events and academic satisfaction in Chinese medical undergraduates

**DOI:** 10.3389/fpubh.2024.1427616

**Published:** 2024-11-22

**Authors:** Jiarun Yang, Xuemei Song, Jili Zhang, Yunge Zheng, Guangyu Chen, Tianyi Bu, Jiawei Zhou, Yuying Tong, Yanjie Yang, Zhengxue Qiao

**Affiliations:** ^1^Department of Psychology, School of Education of Heilongjiang University, Harbin, China; ^2^Psychology and Health Management Center, Harbin Medical University, Harbin, China

**Keywords:** life events, positive coping style, anxiety, academic satisfaction, serial multiple mediating role model

## Abstract

**Objectives:**

Academic satisfaction plays an important role in promoting the future careers of medical undergraduates. Therefore, it is of great significance to improve academic satisfaction by exploring its influencing factors. The purpose of this study was to investigate the serial multiple mediating role of life events, coping styles, anxiety, and academic satisfaction among Chinese medical students.

**Methods:**

In this cross-sectional study, clinical medicine students from a medical university in Heilongjiang Province were surveyed using stratified random cluster sampling procedures. The questionnaires included the Adolescent Life Events Scale, the Simple Coping Style Questionnaire, the Self-rating Anxiety Scale, and the Academic Satisfaction Scale. Pearson’s correlation analysis and bootstrap analysis were used for statistical analysis.

**Results:**

Life events were negatively related to positive coping styles and academic satisfaction and were positively related to anxiety symptoms. Positive coping styles were negatively associated with anxiety symptoms and positively associated with academic satisfaction. Anxiety symptoms were negatively associated with academic satisfaction. The serial multiple mediating role of positive coping style and anxiety in the relationship between life events and academic satisfaction was significant.

**Conclusion:**

The results showed that life events were sequentially associated with decreased positive coping styles and then increased anxiety, which resulted in reduced academic satisfaction among medical students.

## 1 Introduction

The students who receive medical education are generally employed in the medical industry. As a reserve of medical personnel, medical students are a source of strength for the future development of medicine and healthcare in China. In the future, they will innovate medical technology in the medical field, relieve the pain of patients, and promote the development of medicine, which is of great social significance, and this requires medical students to have a high degree of satisfaction with their studies and life at the undergraduate level. This is supported by Lütfi and Yildirim who point out that personal satisfaction during school is one of the most important factors influencing their post-graduation job preferences (1). Academic satisfaction reflects the students’ satisfaction with their studies and the factors that constitute the learning process during college (2, 3). Academic satisfaction plays an important role in the recruitment of new students and the retention and maintenance of continuing education, thus influencing the future development of students and academic institutions (1, 4, 5), and is regarded as a performance evaluation criterion by a large number of higher education institutions (4). Therefore, studying the academic satisfaction of medical students is important to understand their future development and improve the teaching methods of educational institutions.

Given its importance, academic satisfaction has been studied extensively. A study of pharmacy students showed that academic satisfaction was higher in first-and second-year students than in third-and fourth-year students, suggesting that academic satisfaction was higher in the lower grades than in the higher grades (6). One study showed that burnout among nursing students was significantly associated with low academic satisfaction (7). Therefore, in this study, we only discuss the impact of academic satisfaction and do not discuss the part about professional job burnout. Academic motivation and student engagement have been found to be positively related to academic satisfaction (1, 5). Academic satisfaction is also a predictor of career identity (8) and work involvement (9). Students with higher academic satisfaction have higher career identity and work engagement in future jobs. Because of the potentially important role of medical students in the medical field, it is necessary to elucidate the factors influencing academic satisfaction and explore measures to reduce the impact of these factors on medical students.

To date, there have been few studies on the relationship between life events and student satisfaction of medical students, and even fewer studies have focused on the potential mechanism of the relationship between life events and academic satisfaction. Life events, as psychosocial stressors, have been widely studied for their effect on physical and mental health (10). Life events are a series of events or situations that challenge, threaten, damage or overwhelm an individual’s physical or mental capacity (11). They are reflected in family life, work and study, and other aspects of social life, such as academic pressure and interpersonal relationships. A series of life events are always stressful. In today’s competitive environment, students are under more pressure than ever before, whether it is from studies, exams, peers, teachers, or parents (12). Some scholars have conceptualized stressors as academic pressure, interpersonal pressure, and social pressure, all of which have a negative impact on university students’ satisfaction with their educational experience in university (4). These pressures are particularly acute among medical students (13), and studies have pointed out that the high prevalence of stress is one of the most important problems reported among medical students worldwide (14). A study found that perceived stress was negatively correlated with academic satisfaction, implying that the more students who perceived more daily stress, the less satisfied they were with their academic studies (15).

Studies have indicated that coping styles play a mediated role in the relationship between stress and poor mental health outcomes (16). Coping style refers to the way an individual copes with stress, and effective coping styles can help alleviate psychological stress in medical students (17). Depending on their nature, coping styles can be divided into positive coping styles and negative coping styles (18). Both problem-focused and avoidance coping styles were reported to be significant predictors of academic satisfaction (19), and Meneghel et al. found a positive correlation between problem-solving coping styles and academic satisfaction (2). Life events are important sources of psychosocial stress, but coping styles are processes that manage external or internal demands and are important mediating moderators of the psychological stress process (20). Clinical students with appropriate coping methods are more psychologically prepared, especially for the anxiety of caring for pain or facing death, and will have higher scores on their academic satisfaction. Therefore, it is of great significance to explore how life events affect academic satisfaction by influencing coping styles.

Medical students are a high-stress group due to the specific nature of their profession (14, 17, 21), and they also have high levels of anxiety (22, 23). A systematic review of 40 articles related to psychological distress among medical students in the United States and Canada found high levels of anxiety among medical students (22). Elizabeth P. Casline has also shown that more negative life events in academics increase the chances of developing anxiety disorders (24). Anxiety is a common psychological state. As an adaptive emotional response, it has a protective effect (25). However, if the anxiety state becomes systematic, the response may be harmful, producing anxiety disorders (26). Anxiety is also an important factor affecting academic satisfaction. It is well documented that satisfaction with the medical profession and the levels of job stress and burnout associated with performing this profession are conditioned by anxiety and depression, as well as other psychological characteristics (27). The results of a study of pharmacy students showed that anxiety scores were negatively correlated with academic satisfaction scores (15). Vitasari et al. argue that anxiety is detrimental to students’ academic satisfaction (28). Jessica Franzen’s study also showed that lower academic satisfaction scores were closely associated with anxiety (29). Therefore, analyzing the mediating role of anxiety between life events and academic satisfaction can help to further resolve the relationship between life events and academic satisfaction.

The aim of this study was to explore the role of coping style and anxiety in the relationship between life events and academic satisfaction, and we hypothesized that stress from negative life events would be negatively associated with positive coping styles and then positively associated with anxiety, which would be negatively associated with academic satisfaction.

## 2 Methods

### 2.1 Participants

The survey was conducted at a medical university in Harbin City, Heilongjiang Province, China. A total of 735 third-year and fourth-year medical students from two affiliated hospitals of this medical university were selected by stratified random cluster sampling procedures. We randomly selected classes from each year, and all medical students in the selected classes participated in this survey. A self-administered questionnaire was provided to each student by trained investigators. Excluding 42 invalid questionnaires (questionnaires were answered in too short a time or were incomplete), 693 valid questionnaires were finally returned, with a valid recovery rate of 94.28%. Among the 693 medical students, 292 were male students, accounting for 42.1%, and 401 were female students, accounting for 57.9%, with an average age of 21.07 ± 0.79 years old. The study was approved by the Ethics Committee of Harbin Medical University, and all participants gave their written consent.

### 2.2 Measurement of academic satisfaction

The Academic Satisfaction Scale (ASS) was compiled by Hongyu and Rui (30). The ASS consists of 12 items, including three dimensions, and is scored on a 5-point scale. The three dimensions included learning satisfaction, teaching satisfaction, and school hardware satisfaction. Cronbach’s *α* was 0.88.

### 2.3 Measurement of coping style

The Simple Coping Style Questionnaire (SCSQ) compiled by Xie was selected (18). The questionnaire was divided into two dimensions: positive coping styles (items first to 12th) and negative coping styles (items 13th to 20th). Items were assessed on a Likert scale ranging from 0 (no use) to 3 (frequent use). Cronbach’s *α* was 0.90.

### 2.4 Measurement of anxiety

The Self-rating Anxiety Scale (SAS) (31) was developed by WK Zung and consists of 20 questions on a 4-point scale ranging from none to persistent presence of the symptom. The higher the score, the more severe the anxiety. Cronbach’s α was 0.80.

### 2.5 Measurement of life events

The Adolescent Life Events Scale (32) was developed by Liu Xianchen, and it is divided into six dimensions: interpersonal relationships, academic stress, punishment, health adaptation, loss, and others. A 5-point scale is used. The higher the score, the more the life events. Cronbach’s *α* was 0.92.

### 2.6 Statistical analysis

The SPSS package (version 20.0 for Windows) was used to analyze the data. Pearson’s correlation analysis was performed to explore the relationship between life events, positive coping styles, anxiety, and academic satisfaction. In the mediation analysis, sex and age were treated as concomitant variables in the regression analysis; model 6 of the PROCESS macro was used to explore the relationship between the dependent variable (life events) and the dependent variable (academic satisfaction) that may be affected by mediator variable 1 (positive coping styles) and mediator variable 2 (anxiety). After that, the mediated effect was tested by SPSS bootstrap analysis, and 5,000 bootstrap samples were used in this study.

## 3 Results

### 3.1 Academic satisfaction

The results of the descriptive analysis showed that the mean score of medical students’ academic satisfaction was 47.67 ± 6.22, with teaching satisfaction scoring the highest (16.83 ± 2.51) points and hardware satisfaction scoring the lowest (14.31 ± 2.21) points, as detailed in Table 1.

**Table 1:** Mean total score of academic satisfaction and each dimension of medical students’ academic satisfaction.

Learning satisfaction	Teaching satisfaction	School hardware satisfaction	Mean total score
16.52 ± 2.38	16.83 ± 2.51	14.31 ± 2.21	47.67 ± 6.22

### 3.2 Association between psychological capital, organizational commitment, coping styles, and depression

As shown in Table 2, life events were negatively associated with positive coping styles (*r* = −0.144, *p* < 0.01) and academic satisfaction (*r* = −0.213, *p* < 0.01), and life events were positively associated with anxiety symptoms (*r* = 0.575, *p* < 0.01). Positive coping styles were negatively associated with anxiety symptoms (*r* = −0.215, *p* < 0.01) and positively associated with academic satisfaction (*r* = 0.289, *p* < 0.01). Anxiety symptoms were negatively associated with academic satisfaction (*r* = −0.258, *p* < 0.01). The *p*-values between the aforementioned variables were significant, and the correlation coefficient indicated that the effect sizes between the variables were greater than 0.1.

**Table 2:** Correlation between life events, coping styles, anxiety, and academic satisfaction.

Variables	M ± SD	Life events	Positive coping styles	Anxiety	Academic satisfaction
Life events	53.029 ± 18.632	–			
Positive coping styles	25.063 ± 5.931	−0.144**	-		
Anxiety symptoms	40.326 ± 9.352	0.575**	−0.215**	-	
Academic satisfaction	47.665 ± 6.227	−0.213**	0.289**	−0.258**	–

**p < 0.01.

### 3.3 The mediating role of positive coping style and anxiety in the influence of life events on academic satisfaction

**Figure 1:**
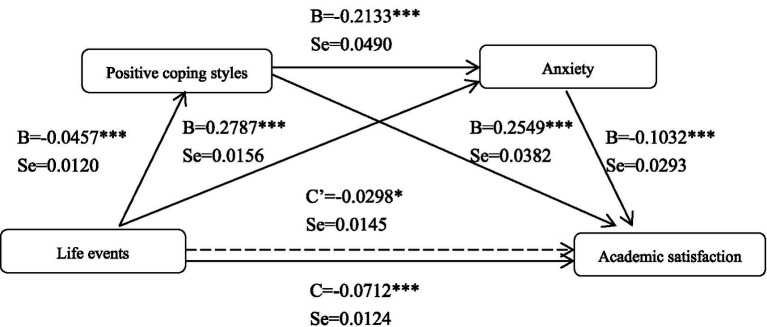
Mediating pathway of academic satisfaction. ***p<0.001.

In this study, academic satisfaction was set as dependent variable Y, life events were set as independent variable X, and positive coping style and anxiety symptoms were set as mediating variables M1 and M2 to conduct mediating effect analysis. The results showed that positive coping style and anxiety played a chain mediating role between life events and academic satisfaction, and the interaction pathway is shown in Figure 1.

The total effect (c = −0.0712, SE = 0.0124, *p* < 0.001) of life events on academic satisfaction was found to be significant. In addition, life events had a negative direct effect on positive coping styles (B = −0.0457, SE = 0.0120, *p* < 0.001) and a positive direct effect on anxiety symptoms (B = 0.2787, SE = 0.0156, *p* < 0.001). The direct effect of positive coping styles as the first mediating variable on the second mediating variable of anxiety symptoms (B = −0.2133, SE = 0.0490, *p* < 0.001) was also found to be significant. A review of the direct effects of the mediating variables on academic satisfaction showed that the effects of positive coping styles (B = 0. 2,549, SE = 0.0382, *p* < 0.001) and anxiety symptoms (B = −0.1032, SE = 0.0293, *p* < 0.001) were significant. When life events and the two mediating variables were simultaneously entered into the model, the direct effect of life events on academic satisfaction was also found to be significant (c’ = −0.0298, SE = 0.0145, *p* < 0.05). Overall, these results revealed that serial multiple mediating role had occurred.

The bootstrap method was then used to test for mediating effects, with 5,000 replicate samples and 95% confidence intervals calculated. The mediating effect results are shown in Table 3. It can be seen that in the pathway from life events, positive coping styles to academic satisfaction, the 95% confidence interval for the positive coping style-mediated pathway was (−0.0208, −0.0044), which did not include 0 and the mediating effect was significant. In the pathway from life events, anxiety to academic satisfaction, the 95% confidence interval for the anxiety-mediated pathway was (−0.0454, −0.0117), which did not contain 0 and the mediating effect was significant. The 95% confidence interval for the life events–academic satisfaction chain-mediated pathway was (−0.0023, −0.0002), which did not contain 0 and the chain mediating effect was significant. Among the three pathways through which life events mediate academic satisfaction, the single effect of positive coping styles accounts for 16.43%, and the single effect of anxiety accounts for 40.45%. The chain mediating effect for both accounts for 1.4%, among which the effect of anxiety is the largest (see Table 3 for details).

**Table 3:** Analysis of the mediating effect of positive coping style and anxiety in the relationship between life events and academic satisfaction among medical undergraduates.

Pathway	Effect value	Boot SE	%	t	P	Bootstrapping 95% BC confidence interval (CI)	
						BootLL CI	BootUL CI
Direct effect	−0.0298	0.0145	41.85%	−2.0484	0.0409	−0.0583	−0.0012
Indirect effect	−0.0414	0.0092	58.15%			−0.0597	−0.0232
X-M1-Y	−0.0117	0.0042	16.43%			−0.0208	−0.0044
X-M2-Y	−0.0288	0.0085	40.45%			−0.0454	−0.0117
X-M1-M2-Y	−0.0010	0.0005	1.40%			−0.0023	−0.0002

M1 = positive coping styles, M2 = anxiety symptoms.

## 4 Discussion

To the best of our knowledge, this is the first study to examine the role of coping style and anxiety in the relationship between life events and academic satisfaction in Chinese medical undergraduates. The results showed that the serial multiple mediating role of positive coping style and anxiety in the relationship between life events and academic satisfaction was significant.

The mean of the total score and the mean of each dimension score of academic satisfaction are greater than the theoretical median of 10 points, which is in the middle to upper level. Students are most satisfied with teaching, followed by learning and finally hardware conditions. Some studies have compared the academic satisfaction of nursing students under the comprehensive curriculum and the traditional curriculum and found that the comprehensive curriculum students have higher satisfaction with curriculum and teaching, while the traditional curriculum students have higher satisfaction with educational environment (7). In our study, the high academic satisfaction scores of medical students were observed to be due to a clear career orientation and involvement in their studies, which, in turn, leads to high academic satisfaction.

### 4.1 The direct effect of life events on academic satisfaction

Medical students face a variety of life events during their undergraduate years, such as immense academic pressure and complex interpersonal relationships, which can cause them distress. The high prevalence of stress is one of the most important issues reported by medical students worldwide (14). This study explored the relationship between life events and academic satisfaction among medical students. The results of the study showed a negative relationship between life events and academic satisfaction among medical students, indicating that life events have a negative impact on academic satisfaction, that is, medical students who experience more life events have lower academic satisfaction. Overall, this finding is consistent with previous studies. A previous international study highlighted the impact of academic stress on students and reported that stress among nursing students prevented them from performing at their best (33), which significantly affected student performance and, in turn, academic satisfaction. Negative life events also take more resources away from students, and they are unable to balance their time and energy to cope with their studies and life well, which, in turn, leads to lower academic satisfaction.

The results suggested that life events not only have a direct effect on students’ academic satisfaction but also have an indirect effect on academic satisfaction through the mediation of coping style and anxiety. There are three crucial pathways through which the mediation model of life events impacts academic satisfaction:

### 4.2 The mediating role of positive coping styles between life events and academic satisfaction

Life events can affect academic satisfaction through positive coping styles. In the pathway from life events, positive coping styles to academic satisfaction, the mediating effect value of positive coping styles is negative. One explanation is that students experience too many life events that multiply their stress, but their coping methods often remain the same (34). In previous studies, positive affect has been shown to promote the use of effective coping styles and enhance problem solving in stressful situations (35). The negative emotions caused by stress cause medical students to become demotivated, less likely to adopt positive coping styles, less interested in learning, and thus less satisfied with their academic performance. Second, experiencing more negative life events can lead to physical problems such as insomnia (14), a poor mental state that makes it difficult to maintain a positive attitude toward life, a lack of personal wellbeing, and a correspondingly low level of academic satisfaction.

### 4.3 The mediating role of anxiety between life events and academic satisfaction

In addition, the study found that life events have an impact on anxiety in the first place, which, in turn, has an impact on academic satisfaction. There was a positive correlation between life events and anxiety, that is, medical students who experienced more negative life events had higher anxiety scores. As reported in previous studies, negative life events were predictive of anxiety symptoms (36, 37). A study of nursing students also indicated that stress in clinical training and practice was associated with increased psychological and physical symptoms (38), providing support for the relationship between stress and anxiety, consistent with the findings of this study. As for the impact of anxiety on academic satisfaction, the results showed a negative correlation between anxiety and academic satisfaction, that is, medical students with high anxiety scores will have lower academic satisfaction. Previous research has also shown that anxiety can have a negative impact on medical students’ academic performance and professional development (22). It is speculated that anxiety may lead to insomnia, memory loss (39), and other problems, and poor learning status leads to low learning satisfaction.

### 4.4 The chain-mediated role of positive coping style and anxiety in the relationship between life events and academic satisfaction

According to our research, life events can affect academic satisfaction through the chain mediation of positive coping style and anxiety. Life events were first negatively related to positive coping styles and then negatively related to anxiety, which, in turn, was negatively related to academic satisfaction.

First, life events were negatively correlated with positive coping styles. This is consistent with the results of previous studies. The series of stressors associated with negative life events can reduce the use of positive coping styles; the study also showed that factors of life events, such as interpersonal relationships, sense of loss, and health adaptation, were negatively associated with positive coping styles (40). In addition, positive coping styles were negatively correlated with anxiety. Anxiety is not directly caused by stressors, but is the result of an individual’s perception of and response to stressors (41) and is influenced by effective coping styles. Denial/acceptance coping styles are greater risk correlates of anxiety (42), whereas students who typically adopt a positive coping style are more resilient and therefore less overly anxious. Furthermore, students with positive coping styles were more likely to get a sense of accomplishment compared with those with negative coping styles, which, in turn, led to a reduction in anxiety. Finally, anxiety is negatively correlated with academic satisfaction. Moderate levels of anxiety related to and triggered by the college campus environment are detrimental to students’ academic performance and satisfaction (15).

Therefore, medical students who actively choose positive ways to cope with stress in general can effectively reduce anxiety and thus achieve higher levels of academic satisfaction. At present, some studies have explored the impact of life events on the mental and emotional health of adolescents and found that the positive coping style plays a partial mediating role between life events and life satisfaction, with the mediating effect accounting for 33.2% of the total effect (34). These results suggest that both life events and coping styles are associated with undergraduate life satisfaction, which is consistent with the findings of our study. Life events are unpredictable, but we can improve their academic satisfaction by guiding medical students to choose more active coping styles. Thus, to improve the academic satisfaction of medical students, medical universities can start from the following aspects.

First of all, psychological education should be actively carried out to develop students’ mental resilience to have enough energy to cope with major life events while popularizing ways to cope with anxiety so that students can help themselves and study with a positive mindset to obtain high academic satisfaction.

Second, the proportion of practical courses should be appropriately increased. Practical classes enable students to grasp knowledge more deeply and firmly, thus improving their academic satisfaction.

Third, different psychological interventions should be implemented for college students of different grades. These interventions should focus on addressing misunderstandings and interpersonal relationships among students, promoting the mental health of college students, and adopting appropriate coping strategies for stressful events.

Finally, psychological screening should be conducted regularly. It can provide timely psychological intervention for students with severe anxiety to keep them in a state of mental health, preparing them for increased academic satisfaction.

## 5 Limitations

Although the study provides a framework regarding the relationship between life events, positive coping styles, anxiety, and academic satisfaction to improve the academic satisfaction and the future development of medical students, there are several limitations in this study. First, the study design was cross-sectional in which there were no clear conclusions about the direction of the causal relationship between life events, positive coping styles, anxiety symptoms, and academic satisfaction. Second, the study only considered the effects of anxiety, without different levels of depression. The research is not comprehensive enough, and the analysis is not broad enough. There are other factors that may contribute to academic satisfaction. Third, the questionnaires were all self-reported, and there may be biases in the recall of data. Finally, the sample was relatively small, the study population was selected from only one medical university, and the generalizability of the findings was limited, without considering the regional gap.

## 6 Conclusion

This is the first study to examine the role of coping style and anxiety in the relationship between life events and academic satisfaction among Chinese medical undergraduates. The results of the present study showed that life events were sequentially associated with decreased positive coping styles and then increased anxiety, which resulted in reduced academic satisfaction among medical students. Effective measures for improving the academic satisfaction of medical students should be adopted.

## Data Availability

The raw data supporting the conclusions of this article will be made available by the authors, without undue reservation.
